# Virus Taxonomy: One Step Forward, Two Steps Back

**DOI:** 10.3201/eid1001.030945

**Published:** 2004-01

**Authors:** Mark Eberhard

**Affiliations:** * Centers for Disease Control and Prevention, Atlanta, Georgia, USA

**Keywords:** Taxonomy, virus, virology

Taxonomy, the science of identifying and naming entities, has long been an integral component of biologic sciences, both in botany and zoology. Not all biologists are actively engaged in taxonomy (only a few truly enjoy working in this field), yet everyone, even lay persons, recognizes the value of consistency and standardization in the naming of animate and inanimate objects. The naming of biologic entities is an exact tool that conveys a precise meaning and ensures maximal continuity and universality for present and future generations. It further confirms that when reference is given to a scientific name, such as *Quercus* *albus* or *Gorilla gorilla beringei*, everyone recognizes we are speaking about white oak trees or mountain gorillas, respectively, and not northern red oak trees (*Quercus* *rubra*) or lowland gorillas (*Gorilla gorilla* *gorilla*). However, taxonomy has not been, and still is not, without difficulties. Very early on, it was recognized that universal “codes” needed to be developed to guide the naming of biologic entities so that each name would be as unique and distinct as the object being named.

 Virologists seem to be struggling with taxonomy more than scientists in other disciplines. Much of this struggle seems self-induced, and the article by van Regenmortel and Mahy, “Emerging Issues in Virus Taxonomy”(*1*), continues to provide highly controversial reading for those who might have an interest in how to approach taxonomic issues. For taxonomists and other scientists with a strong sense of historical perspective, that article may be difficult reading. The article illustrates the inconsistencies between viral taxonomy and taxonomy of other biologic disciplines.

The authors do continue to chip away at several fundamental issues, and they are to be commended for that. For instance, the authors acknowledge that viruses are biologic entities, they advocate applying species names in virus taxonomy, and they recognize that use of a binomial naming system is preferred. That virologists also recognize the value of using a combination of characters to define a species is not novel but reflects that elements other than morphologic features, i.e., host and geographic distribution, vector requirements, and molecular sequences, contribute to defining a species. Although seemingly at the very core of taxonomy, agreement on such basic principles is a major step, when one considers that not all virologists subscribe to standard biologic principles.

 As the authors note, virus taxonomy is an emerging discipline that allows working virologists to communicate without misunderstanding. However, the issue is larger than that, and virologists need to know that they are not only working to solve a problem in virology, but they are also accountable to the larger biologic community so that we can communicate clearly and effectively across disciplines. Only with consistency and uniformity will this communication occur. The overall goal should be to provide consistency not only within the field of virology, but also and more importantly, across the broader field of biology. In a recent article by Ashford, the need for consistency in defining terms was noted, and the author stated, “When we all agree on what we are talking about, we will understand each other better” (*2*). Whether dealing with definition of terms or taxonomic categories, consistency across fields is paramount.

 This is where this article (and seemingly most efforts to date on virus taxonomy) falls short. Inconsistencies in virus taxonomy—some perpetuated in this article, some introduced in it, indicate that, unless more attention is paid to what has gone before, virus taxonomy will never achieve the respect it deserves. For instance, insistence on italicizing names above genus level is out of character with most other zoologic disciplines. Similarly, the idea that viruses are unique and need their own set of rules appears presumptuous. All biologic entities are unique: humans are unique, a particular bacterium is unique, as are specific parasites, plants, algae, and the like. Viruses are different and distinct but occupy a spot along a continuum in the bigger biologic spectrum. Prions may be even more problematic than viruses to characterize and name.

The most perplexing proposal offered in this article is the placement of the genus name after the species name. Taxonomy, and the more specific aspect of nomenclature, is based on a traditional system of naming organisms beginning with the highest order (kingdom) and descending to lowest order (species). Why virologists would wish to be discordant with the rest of biologic science is unclear, and no immediate value to such a system is evident. Virologists also need not worry about having a universally acceptable definition of a species before applying rigorous rules of taxonomy. Every discipline, botanical and zoologic, is wrestling with a working definition of what constitutes a species. Virologists should also not fret over whether a species concept only applies to sexually reproducing organisms. Other biologists are not so encumbered, as the International Code for Zoological Nomenclature (*3*) and the International Code of Botanical Nomenclature (*4*) very nicely handle plants, fungi, bacteria, and protozoa that reproduce asexually. This point is further supported by the fact that even newly discovered fossil plants and animals are routinely named according to rules found in these codes, and it can be stated with a fair degree of certainty that most fossils have not engaged in sexual activity for centuries, if not millennia.

 Other troubling areas of current taxonomy of viruses include the concept that viruses are abstract entities. How comforting will it be to patients with serious diarrhea caused by Norwalk virus to learn that their illness is caused by an abstract entity? Viruses cause disease just as parasites and bacteria cause disease, and we do not consider them abstract. Because parasites or bacteria are different from viruses in composition, life cycle, and the like does not make a virus an abstract entity. Developing scientific names for viruses has also provided some humorous fodder for other taxonomists. Using English rather than latinized words for names appears capricious and is dismissive of centuries of distinguished scientists and pioneers in the field, including virology. Lastly, virologists’ concern about having to demarcate and coin new names for an estimated 1,550 virus species is puzzling. One wonders what their response would be to naming and cataloguing in other disciplines, such as entomology, where there are >1 million recognized species, some 10,000 new species described each year, and an estimated 4–6 million species yet to be discovered and named (*5*,*6*).

 From the perspective of a nonviral taxonomist, virologists might do well not to reinvent the wheel (*7*) but rather to adopt and use existing, conventional taxonomic structure, as in the International Code of Zoological Nomenclature (*3*). The rules, concepts, and framework have all been worked out, have been tested over time, and, best of all, are immediately available for use.

 Drs. van Regenmortel and Mahy are to be commended for trying to bring virus taxonomy to a higher order of consistency. However, given the controversies, virus taxonomy may not get it right (*8*) for some time. This situation is unfortunate as there is an increasing need, as recently evidenced by the severe acute respiratory syndrome outbreak, to detect, study, develop effective treatments for, and ultimately control and prevent viral infections. Virus taxonomy should become a stabilizing force, rather than a distraction, during these challenging times.

## References

[1] van Regenmortel M, Mahy B. Emerging issues in virus taxonomy. Emerg Infect Dis. 2004;10:8–13. 15078590 10.3201/eid1001.030279 PMC3322749

[2] Ashford R. When is a reservoir not a reservoir? Emerg Infect Dis. 2003;9:1495–6. 14725261 10.3201/eid0911.030088 PMC3035539

[3] International Code of Zoological Nomenclature. (4th ed). International Trust for Zoological Nomenclature, London, England, 2000. Also available from: URL: http://www.iczn.org/code.

[4] International Code of Botanical Nomenclature (St. Louis code). Regnum Vegetabile 138. Konigstein, Germany: Koeltz Scientific Books; 2000.

[5] Insects. Columbia encyclopedia, 6th ed. [accessed on 11/9/03]. Available from: URL: http://www.encyclopedia.com

[6] Kirby A. Insect species ‘fewer than thought.’ BBC News. [accessed on 11/9/03]. Available from: URL: http://news.bbc.co.uk/1/hi/sci/tech/1949109.stm

[7] Eberhard M. Letters to the editor. Am J Trop Med Hyg. 2003. In press.

[8] Calisher C, Mahy B. Taxonomy: get it right or leave it alone [editorial]. Am J Trop Med Hyg. 2003;68:505–6. 12812333 10.4269/ajtmh.2003.68.505

